# Cognitive Trajectories in Older Patients with Cancer Undergoing Radiotherapy—A Prospective Observational Study

**DOI:** 10.3390/curroncol29070409

**Published:** 2022-07-21

**Authors:** Guro Falk Eriksen, Jūratė Šaltytė Benth, Bjørn Henning Grønberg, Siri Rostoft, Øyvind Kirkevold, Sverre Bergh, Anne Hjelstuen, Darryl Rolfson, Marit Slaaen

**Affiliations:** 1 The Research Center for Age-Related Functional Decline and Disease, Innlandet Hospital Trust, 2313 Ottestad, Norway; j.s.benth@medisin.uio.no (J.Š.B.); oyvind.kirkevold@aldringoghelse.no (Ø.K.); sverre.bergh@sykehuset-innlandet.no (S.B.); marit.slaaen@sykehuset-innlandet.no (M.S.); 2 Department of Internal Medicine, Hamar Hospital, Innlandet Hospital Trust, P.O. Box 4453, 2326 Hamar, Norway; 3 Institute of Clinical Medicine, Faculty of Medicine, University of Oslo, Pb 1171 Blindern, 0318 Oslo, Norway; srostoft@gmail.com; 4 Institute of Clinical Medicine, Campus Ahus, University of Oslo, P.O. Box 1171, 0318 Blindern, Norway; 5 Health Services Research Unit, Akershus University Hospital, P.O. Box 1000, 1478 Lørenskog, Norway; 6 Department of Clinical and Molecular Medicine, Norwegian University of Science and Technology (NTNU), NO-7491 Trondheim, Norway; bjorn.h.gronberg@gmail.com; 7 Department of Oncology, St. Olavs Hospital, Trondheim University Hospital, NO-7491 Trondheim, Norway; 8 Department of Geriatric Medicine, Oslo University Hospital, Pb 4956 Nydalen, 0424 Oslo, Norway; 9 The Norwegian National Centre for Ageing and Health, Vestfold Hospital Trust, Postboks 2136, 3103 Tønsberg, Norway; 10 Faculty of Health, Care and Nursing, NTNU Gjøvik, P.O. Box 191, N-2802 Gjøvik, Norway; 11 Department of Internal Medicine, Innlandet Hospital Trust, Kyrre Grepps gate 11, 2819 Gjøvik, Norway; anne.hjelstuen@sykehuset-innlandet.no; 12 Division of Geriatric Medicine, University of Alberta, Edmonton, AB T6G 2R3, Canada; drolfson@ualberta.ca

**Keywords:** Montreal Cognitive Assessment, cancer-related cognitive impairment, geriatric oncology, cognitive function, physical impairment, frailty

## Abstract

Cognitive function can be affected by cancer and/or its treatment, and older patients are at a particular risk. In a prospective observational study including patients ≥65 years referred for radiotherapy (RT), we aimed to investigate the association between patient- and cancer-related factors and cognitive function, as evaluated by the Montreal Cognitive Assessment (MoCA), and sought to identify groups with distinct MoCA trajectories. The MoCA was performed at baseline (T0), RT completion (T1), and 8 (T2) and 16 (T3) weeks later, with scores ranging between 0 and 30 and higher scores indicating better function. Linear regression and growth mixture models were estimated to assess associations and to identify groups with distinct MoCA trajectories, respectively. Among 298 patients with a mean age of 73.6 years (SD 6.3), the baseline mean MoCA score was 24.0 (SD 3.7). Compared to Norwegian norm data, 37.9% had cognitive impairment. Compromised cognition was independently associated with older age, lower education, and physical impairments. Four groups with distinct trajectories were identified: the very poor (6.4%), poor (8.1%), fair (37.9%), and good (47.7%) groups. The MoCA trajectories were mainly stable. We conclude that cognitive impairment was frequent but, for most patients, was not affected by RT. For older patients with cancer, and in particular for those with physical impairments, we recommend an assessment of cognitive function.

## 1. Introduction

Cognitive impairment is a frequent problem in older age. Among patients with cancer ≥65 years, approximately 3.8–7% have dementia [1], and cognitive impairment is reported in 36% of patients over 70 years with advanced cancer [2]. Over the last decade, there has been an increasing awareness of a condition referred to as cancer-related cognitive impairment (CRCI) [3,4,5,6,7]. CRCI is characterized by a patient-reported and objectively measured cognitive decline presenting in relation to cancer and/or its treatment [4]. Several studies suggest that older patients with cancer, and especially frail older patients [8], are at particular risk of experiencing a decline in cognitive function during systemic cancer therapy [9,10,11]. This is concerning, as older patients with severe and life-limiting disease consider preserved cognitive function as one of the most important treatment outcomes [12].

CRCI has mainly been studied in women receiving adjuvant chemotherapy for breast cancer [4], and the phenomenon was for some time referred to as “chemobrain” [13]. More recently, it has been advocated that this term is misleading because the condition probably has a more complex underlying etiology [13]. In addition to issues that are common among patients with cancer and are known to affect cognitive function, such as comorbid conditions, polypharmacy, and depression, frequently occurring symptoms, including fatigue and treatment modalities other than chemotherapy, could also be important influencing factors [3,9,13,14]. There are indications that endocrine therapy can contribute to CRCI in patients with breast and prostate cancer and that immunotherapy and antiangiogenics can have a negative impact [3,7]. Except for research on patients with childhood cancer and tumors involving CNS [15], little is known about how radiotherapy (RT) affects cognitive abilities [6,7].

The assessment of cognitive function is not routinely performed in oncology practice. Hence, cognitive impairment may easily be overlooked [16,17]. Cognitive impairment can have several important implications. It can influence patients’ treatment preferences, shared decision making, treatment adherence, the reporting of toxicities, and self-care abilities [18]. Therefore, the evaluation of cognitive function is an important part of a geriatric assessment (GA) and is recommended in all oncology settings [19,20]. The Montreal Cognitive Assessment (MoCA) test was developed as a screening tool to detect the symptoms of mild cognitive impairment (MCI) [21]. The test is sensitive when applied to older adults with cancer [22] and is a recommended by the leading organizations in the field [14,20,23,24,25].

We previously showed that the age-related health issues identified by GA impact overall survival, quality of life, and physical function in a cohort of older patients with cancer receiving RT [26]. In the present study, addressing the same cohort, our aim was threefold. First, we aimed to describe the prevalence of cognitive impairment by comparing patients’ MoCA scores to Norwegian normative data. Second, we explored the associations between MoCA scores and predefined cancer-related factors assumed to have an impact on cognitive function. Third, we intended to study the development of cognitive function during the course of RT, seeking to identify groups with distinct MoCA score trajectories.

## 2. Materials and Methods

### 2.1. Patients

From February 2017 to July 2018, we conducted a prospective, single-center, observational study at the radiotherapy unit (RTU) of a Norwegian local hospital serving approximately 370,000 inhabitants [27]. Details about the study design, setting, and conduct have been described [26]. The inclusion criteria were referral for RT with curative or palliative treatment intent, age ≥ 65 years, histologically confirmed malignant disease, residence in the hospital catchment area, fluency in oral and written Norwegian, and a capacity to answer self-report questionnaires. The municipal home-care services in 41 of 48 municipalities in the hospital catchment area committed to allocate a designated cancer contact nurse to perform patients’ evaluations during follow-up.

### 2.2. Assessments

Baseline sociodemographic and medical data were attained through patients’ interviews, supplemented by their electronic medical records. The collected data included age, gender, educational level, Eastern Cooperative Oncology Group performances status (ECOG PS) (dichotomized 0–1 or 2–4), cancer diagnosis (grouped as breast, prostate, lung, or other types of cancer), previous cancer treatment, RT regimen, and treatment intent (curative or palliative). Patients answered the European Organisation for Research and Treatment of Cancer Quality-of-Life Core Questionnaire version 3.0 (EORTC) (QLQ-C30) [28], which includes three items assessing fatigue. These items are scored from 1 (not at all) to 4 (very much), and before analyses, raw scores are converted to a fatigue scale ranging from 0–100 [29]. Higher scores indicate more fatigue. At baseline, patients underwent a modified geriatric assessment (mGA) [26], including an evaluation of comorbidities (Charlson Comorbidity Index (CCI) [30]) and polypharmacy (number of daily medications), depression (Geriatric Depression Scale-15 (GDS-15) [31]), and physical domains, i.e., nutritional status (Mini Nutritional Assessment Short Form (MNA-SF), scored 0–14 [32]), mobility (Timed Up and Go (TUG), measured in seconds [33]), falls (number of falls the last six months), basic activities of daily living (ADL) (Barthel Index, scored 0–20 [34]), and instrumental ADL (IADL) (Nottingham Extended Activities of Daily Living (NEADL), scored 0–66 [35]). Based on well-established and/or commonly used reference values, and as elaborated in a previous publication [26], cut points for impairment in physical domains were defined as Barthel Index score <19, NEADL score < 44, ≥2 falls the last six months, TUG ≥ 14 s, and MNA-SF scores ≤ 11 (at risk of malnutrition). For the purpose of the present paper, we summarized the number of physical impairments for individual patients. Cognitive function was assessed by the MoCA test [21], Norwegian version 7.1, as part of an mGA. The test takes about 10 min to complete and assesses cognitive functions with scores for the following items: visuospatial abilities, the naming of objects, attention and concentration, language, abstraction, working memory, and orientation to time and place [36]. All scores are summarized 0–30 points, with higher scores indicating better function. One extra point is added for persons with ≤12 years of education up to a max score of 30. A difference in MoCA score of ≥3 points (10%) is considered a clinically significant difference [22]. The MoCA test was applied at four time points: at baseline (T0), at RT completion (T1), and eight (T2) and 16 (T3) weeks after completing RT. Per the protocol, the T1 assessment was omitted for patients receiving ≤9 RT fractions. For these patients, the interval between T0 and T1 would be less than two weeks, which we considered too short to detect any clinically meaningful change in MoCA scores. The T2 and T3 assessments were not performed for patients residing in non-committing municipalities. A study nurse or a resident physician in oncology performed the tests at T0 and T1 at the RTU. Subsequent tests were performed by a municipal cancer contact nurse at the patients’ current residences. All test personnel received the same specific training in addition to a manual with detailed scoring instructions. If the results of the tests at T2 and T3 were not received within a week after the scheduled assessment, the municipal cancer contact nurse received a reminder.

### 2.3. Statistical Approach

Our statistical approach was descriptive and explorative. Categorical data were described with frequencies and percentages, and continuous data were described with means and SDs or medians and min–max values. To compare characteristics between groups of patients, a Student’s *t*-test, ANOVA, or χ^2^-test was applied, as appropriate. Using a publicly available MoCA score calculator [37], the baseline MoCA scores were compared to Norwegian normative data from a population of community-dwelling adults aged ≥70, excluding those with a history of dementia, mild cognitive impairment, stroke, or depression [38]. The MoCA calculator provides the person’s Z-score, i.e., the number of SDs from the mean normative MoCA score, accounting for educational level, age, and gender. MoCA scores more than 1 SD below the age-, education-, and gender-matched Norwegian norm were used to define cognitive impairment [37]. The patients included in the present study aged 65–69 years were, for these specific analyses, assigned the age of 70 years. For descriptive purposes, we also estimated the proportion of patients with MoCA scores below 26, which is the originally suggested cut point for mild cognitive impairment [21]. Unadjusted and adjusted linear regression models were estimated to assess the association between baseline MoCA scores and predefined cancer-related factors of potential importance. These factors were previous cancer treatment (categorized as endocrine therapy, other systemic therapy (including chemotherapy), cancer surgery, and/or RT), RT treatment intent (curative or palliative, reflecting disease stage, brain cancer, or brain metastases), and fatigue (patient-reported on the QLQ-C30), in addition to a number of physical impairments (continuous 0–5 ADL, IADL, falls, mobility, and nutritional status). The model was adjusted for factors known to influence cognitive function, i.e., age, gender, educational level (categorized as completed compulsory (≤10 years), secondary (11–13 years), or college or university (≥14 years) education), comorbidity (CCI scored 0–26), medications (number of daily mediations), and depression (GDS ≥ 5) [3,4,6,7,38]. Only one patient had been diagnosed with dementia according to CCI. Hence, dementia diagnosis was not taken into account. Spearman’s rho was calculated among all predefined variables. However, no multicollinearity issues were identified (Supplementary Table S1). A growth mixture model was estimated to identify unobserved groups of patients following distinct MoCA score trajectories. The optimal number of groups was determined using a Bayes information criterion, where a smaller value means a better model, backed by the requirement of reasonably large groups, average within-group probabilities larger than 0.8, and non-overlapping 95% confidence intervals (CIs) for trajectories. For sensitivity analyses, we estimated two growth mixture models identical to the one described above. The first excluded patients who died during the 16-week follow-up, and the second included only patients who completed MoCA at all four time points. All tests were two-sided, and results with *p*-values below 0.05 were considered statistically significant. The analyses were performed in SAS v9.4 and STATA v16.

### 2.4. Ethical Considerations

All patients provided written informed consent. The patients’ capacity to consent was evaluated and confirmed by the treating oncologist. If the assessments revealed previously unrecognized severe health problems, test personnel followed pre-defined guidelines for actions. The study protocol was approved by the Regional Committee for Medical Research Ethics South East Norway (protocol code 2016/2031, approved 16 January 2017), and was registered at clinicaltrials.gov (NCT03071640).

## 3. Results

### 3.1. Study Recruitment and Patient Characteristics

During the recruitment period, 301 (59.1%) eligible patients were enrolled. Reasons for non-inclusion were refusal to participate (148 (29.1%])), being considered too sick (28 (5.5%)), and other (e.g., absence of a study nurse) (32 (6.3%)). A total of 298 patients completed the baseline MoCA test and were included in the present study. The mean age was 73.6 years (SD 6.3), and 141 (47.3%) were female. Most patients had completed Norwegian compulsory education (age 6–16) (30.3%) or secondary school (age 16–19) (40.4%), 162 (54.4%) received RT with curative intent, and 16 (5.4%) had brain cancer or brain metastases (Table 1). One physical impairment was found for 99 (33.6%) patients, while 86 (29.2%) had two or more. Additional details on previous cancer treatment and mGA results are displayed in Table 1. Furthermore, 255 (85.6%) had ECOG PS 0-1, and the distribution of cancer diagnoses was 95 breast (31.9%), 73 prostate (24.5%), 63 lung (21.1%), and 67 (22.5%) had other types of cancer. The median number of RT fractions was 14.8 (1–39), and the median dose was 40.0 (4.0–78.0) Gray. Only one patient resided in a nursing home, while 286 (96%) lived in their own residence, either alone (102, 34.6%) or with their spouse/children/others (195, 65.4%).

### 3.2. MoCA Completion Rates, Scores, and Comparison to Norwegian Normative Data

**Figure 1:**
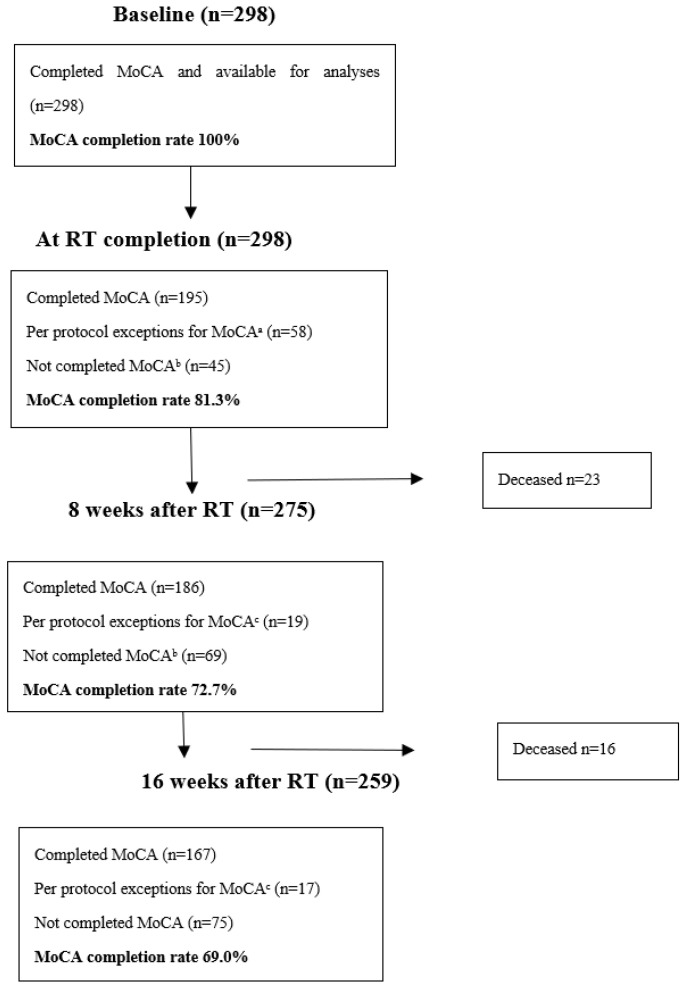
Patient flow chart and MoCA completion rates. ^a^ Patients receiving ≤9 fractions, per protocol, did not perform the MoCA test at the time of RT completion. ^b^ Excluding per protocol exceptions and deceased patients. ^c^ Patients alive at time of assessment and recruited from municipalities that did not participate in performing the mGA during follow-up.

Within 8 and 16 weeks after RT completion, 23 and 39 patients had died, respectively. Accounting for deaths and per protocol exceptions, the MoCA test completion rates at T1, T2, and T3 were 81.3%, 72.7%, and 69.0%, respectively (Figure 1).

The mean baseline MoCA score was 24.0 (SD 3.7, (min–max 10–30)). At T1, T2, and T3, the mean MoCA scores were 25.6 (SD 3.7), 26.3 (SD 4.4), and 27.1 (SD 3.3), respectively. The most frequently impaired MoCA domains at baseline were working memory (91.9%), abstraction (59.1%), visuospatial abilities (65.1%), and language (68.1%) (Table 2).

**Figure 2:**
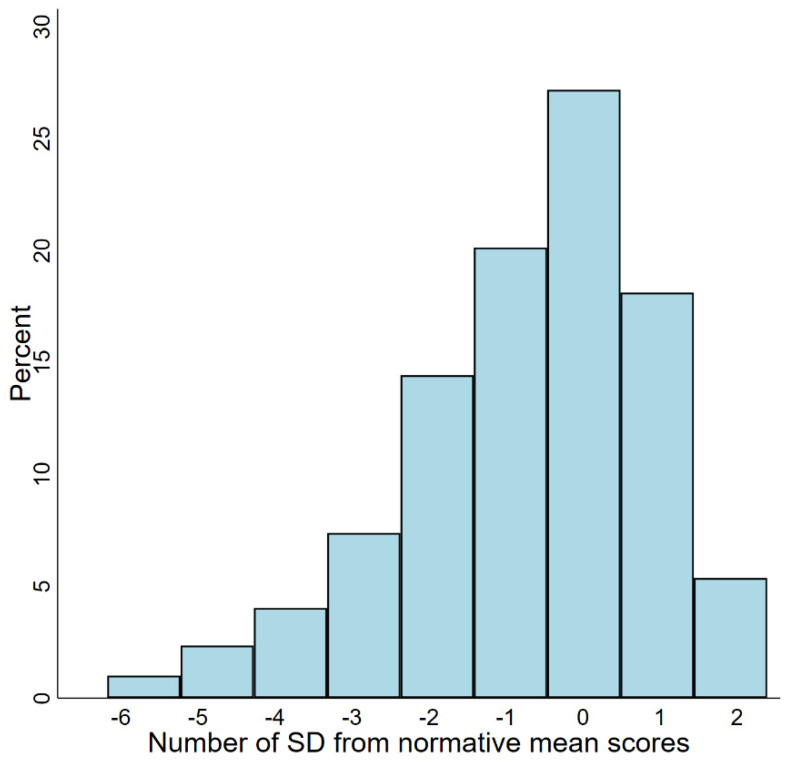
Distribution of MoCA z-scores (SD) based on Norwegian normative data.

According to the recommended MoCA score cut-off at 26 points, 186 (62.4%) had mild cognitive impairment. Compared to Norwegian normative data, 107 (35.9%) patients had MoCA scores 1–2 SDs above the mean, and 78 (26.2%) had scores <1 SD below the mean. In sum, 185 (62.1%) had scores within what is considered the normal range or better (Figure 2). A total of 113 (37.4%) patients had MoCA scores more than 1 SD below the normative mean, indicating cognitive impairment. Among these, 61 patients (20.5% of the overall cohort) had scores more than 2 SDs below the mean.

Comparing completers and non-completers at T3 (Table 3), we found that, at the time of inclusion, non-completers had poorer MoCA scores, used more daily medications, and had more physical impairments and fatigue.

Moreover, a higher proportion had received systemic therapy (including chemotherapy and excluding endocrine therapy), had cancer affecting the brain, and were treated with palliative intent (Table 3). These differences were larger between completers and non-completers due to death than completers and alive non-completers (analyses not shown). The reasons for non-completion were not registered at T1. For the non-completers still alive at T2 (*n* = 69) and T3 (*n* = 75), the reasons for missing the test were related to the home-care services (not enough time and a shortage of nurses at disposal) in 11 and 11 cases, respectively, and to the patients’ condition (too ill/admitted to hospital, did not want to perform the test) in 26 and 29 cases, respectively.

### 3.3. Factors Associated with Baseline MoCA Scores

The results of the linear regression models assessing the impact of predefined variables on baseline MoCA scores are presented in Table 4.

According to unadjusted models, all covariates except gender, cancer affecting the brain, and previous systemic cancer treatment were significantly associated with baseline MoCA scores. In the adjusted model, a higher number of physical impairments (regression coefficient (RC) −0.82, 95% CI [−1.16; −0.48]) and increasing age (RC −0.13, 95% CI [−0.19; −0.07]) remained associated with lower MoCA scores, whereas college/university as compared to compulsory education was associated with higher MoCA scores (RC 2.41, 95% CI [1.50; 3.33]).

### 3.4. MoCA Score Trajectories

**Figure 3:**
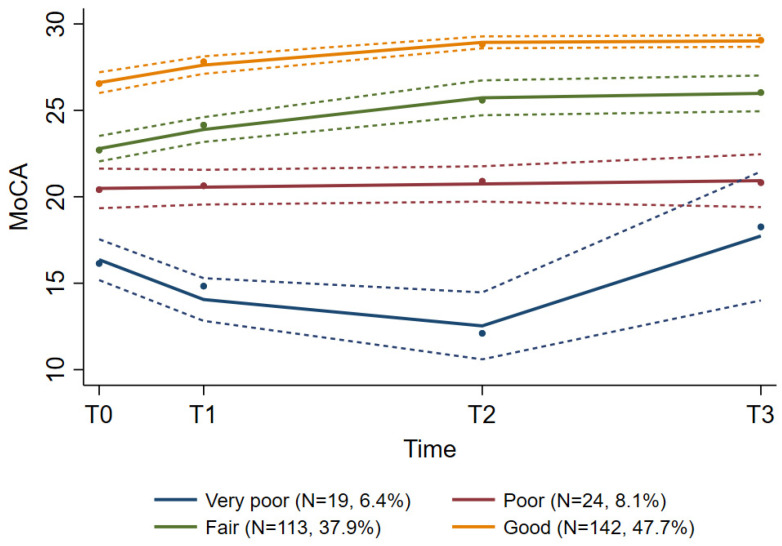
Groups with distinct MoCA score trajectories during the course of radiotherapy.

A growth mixture model identified four groups of patients following distinct MoCA score trajectories, which we named very poor (*n* = 19, 6.4%), poor (*n* = 24, 8.1%), fair (*n* = 113, 37.9%), and good (*n* = 142, 47.7%) (Table 5, Figure 3). The average group probabilities varied between 0.79 (fair group) and 0.91 (good group), and the 95% CIs were non-overlapping, indicating homogeneous groups. For the small group with very poor scores, a clinically significant (≥3 points) transient decline in MoCA scores from T0 to T2 was registered, followed by an improvement beyond pre-treatment levels at T3. The fair group experienced a significant improvement in MoCA scores from T0 to T3. The other two groups had relatively stable trajectories. The patient characteristics of these four groups are presented in Table 1. The proportion of patients with advanced age and with a higher number of physical impairments, comorbidities, and daily medications gradually increased from the good group to the very poor group, whereas the proportion with higher education gradually decreased (Table 1). Our first sensitivity analysis, excluding all patients who died within 16 weeks after RT (T3), reproduced the results of the main analysis. The small “very poor” group consisted of 9 patients (10 out of 19 patients in this group died) with a decline from baseline to eight weeks after RT, followed by an improvement (data not shown). The compliance in this small group was poor, even when those who died were excluded, i.e., at T0 all nine patients completed MoCA, at T1 and T2 six patients completed, whereas at T3 only two patients were completers. In our second sensitivity analysis, only including patients who completed MoCA at all time points (*n* = 113), we also identified four groups with distinct MoCA trajectories ranging from good to very poor (Supplementary Figure S1 and Supplementary Table S2). Similar to the results of the preceding analyses, the trajectories of the good, fair, and poor group were mainly stable. For the very poor group (*n* = 2), however, a significant improvement was registered, demonstrating that the improvement observed at T3 in the “very poor” group, identified in both the main analysis and the first sensitivity analysis, could be attributed to these two patients only.

## 4. Discussion

In this study, we have shown that cognitive impairment was frequent in a heterogeneous cohort of older patients undergoing RT. Age, lower education, and physical impairments were independently associated with compromised cognition. We identified four groups of patients with distinct non-overlapping trajectories of MoCA scores. The majority had stable trajectories, but for the group with the poorest overall cognitive function, a declinewas registered.

To our knowledge, this is the first study to longitudinally assess objective cognitive function in older patients with cancer receiving RT and the first to use the MoCA test for this purpose. According to the original recommended cut-off value at <26 points, assigning one extra point to all with ≤12 years of education [21], the prevalence of cognitive impairment was high (62.4%). However, several studies have indicated that this cut-off may be too high [39,40,41,42,43] and that MoCA scores, in addition to education, could be dependent on age, gender, and cultural aspects. Therefore, we chose the recommended approach [44] and compared patients’ scores with recently published Norwegian normative data [38]. According to this, 37.9% of our patients had MoCA scores consistent with cognitive impairment. Thus, our findings support the view that when using a more stringent MoCA score cut-off, the prevalence of cognitive impairment could be overestimated [39,40,41,42,43]. Nevertheless, we find the prevalence of cognitive impairment among older patients with cancer referred to RT alarming, in particular as 20.5% had MoCA scores more than 2 SDs below the normative mean, which indicates dementia [38]. The high prevalence of cognitive impairment among study participants is consistent with studies indicating that CRCI is a common and underdiagnosed problem among older patients [2,3,17]. In line with other reports [3,44], we also found that executive functions, memory, and attention were the cognitive domains that were most frequently impaired.

According to our adjusted regression model, age, educational level, and the number of physical impairments were the only factors independently associated with baseline MoCA scores. That higher age and lower education negatively affect MoCA results is well-known from several studies [38,40,43]. Physical impairments are indicators of physical frailty [45], and the association between physical frailty and cognitive impairment has been widely confirmed [46,47,48,49]. Opposed to our expectations and a smaller pilot study on early breast cancer [50], previous treatment with systemic cancer agents was not significantly associated with poorer cognition in the adjusted or unadjusted models. However, in our study, about 50% had advanced cancer (palliative treatment intent), which is found to be associated with reduced cognitive function, even before the initiation of systemic therapy [51]. Furthermore, the majority had previously received several treatment modalities. Thus, as concluded by the authors of a study reporting no difference in cognitive decline between women ≥65 years receiving and not receiving adjuvant chemotherapy [11], the observed decline could be attributed to the joint effect of the cancer and the overall treatment burden, making it impossible to disentangle the impact of one treatment from another. In contrast to the established knowledge [15], we also found no association between MoCA scores and cancer affecting the brain. This is most likely explained by the very small number of patients in this subgroup of our cohort. Gender was another factor that had no association with cognitive function in the adjusted and unadjusted models. Although this is in line with reports from other countries [42], the finding contrasts a study of a Norwegian cognitively healthy population ≥70 years, showing that women aged 70–74 years with education of >13 years had the best MoCA scores [38]. It is possible that the severity of other conditions among our patients masked a potential impact of gender. In line with previous reports, comorbidity, medications, depression, and fatigue were significantly associated with cognitive function in unadjusted models [3,4,6,7]. As these associations disappeared in the adjusted model, it might be an indication that the association between MoCA and these factors is weaker than between MoCA and age, education, and the number of physical impairments.

Four groups with distinct MoCA trajectories were identified, varying from good to very poor. The differences in cognitive function between groups persisted from baseline throughout the follow-up period. Moreover, we observed a higher proportion with poorer health, including more comorbidities, daily medications, and physical impairments, from the good to the very poor group. Thus, the identified groups may be seen as representing a continuum from robust to frail, and this finding is in line with other studies suggesting that frailty may be associated with compromised cognitive function [47,52] that might further be negatively affected by cancer and its treatment in older adults [3,8,9,52,53,54]. A wide range of mechanisms explaining this phenomenon have been proposed, including DNA damage, inflammation, and oxidative stress [4,6,7,53]. Similarly, systemic inflammation due to RT has been hypothesized to impair cognition, but existing evidence is very limited [6,55]. In our cohort, the majority had stable or improved cognitive trajectories. This is consistent with previous research in older adults that indicated that RT tolerance is generally good [56,57]. The decline in MoCA scores observed in the small group with the poorest trajectory and poorest health, where only two patients completed MoCA at T3, may be attributed to frailty and reduced cognitive reserves [4]. This assumption is supported by our comparison of the baseline characteristics between completers and non-completers at week 16 after RT, showing that non-completers had poorer cognitive and physical health. The improvement from 8 to 16 weeks after RT reflects the results of two patients, as demonstrated by our sensitivity analysis, and must be interpreted accordingly. However, the overall trajectory of the very poor group should be interpreted with caution due to the small number of patients and substantial attrition. Attrition might also explain the improvement in cognitive function in the fair group, but as this was less pronounced, it is more likely that the transient distress and attention deficits in connection with the start of a new treatment may have affected baseline MoCA scores negatively.

Our results add to the growing evidence showing that multiple factors can contribute to cognitive impairment among older patients with cancer, with age, education, and physical impairments being the most essential. A pre-treatment cognitive assessment is important among older adults, and patients with physical impairments need special attention. As cognitive impairment is associated with negative outcomes such as increased chemotherapy toxicity [20], reduced survival [22], dependency, and reduced quality of life [49], supportive measures before, during, and after RT are necessary.

The strengths of this study are the prospective design, the relatively large sample size, and the mGA performed at baseline. The use of MoCA to assess cognitive function is also a strength in a longitudinal study. In addition to being a sensitive screening tool among older adults in general and older patients with cancer in particular [22], MoCA is reliable in detecting changes in cognitive function over time [58]. Furthermore, the MoCA completion rate was high at all assessment points, and all health care professionals conducting MoCA received the same training. Finally, in the absence of universally accepted and applicable MoCA cut points for cognitive impairment, it is a considerable strength that patients’ scores were compared to Norwegian normative data. Besides attrition, as discussed above, this study has some limitations. Representing mean values, our results reflect MoCA scores on group level, and it should be kept in mind that individual trajectories may occur within the groups. The cohort is heterogeneous in terms of cancer diagnoses and disease stages, and the results may not be applicable to specific groups of patients. However, this could also be regarded as a strength since this reflects the heterogeneity among patients seen in routine clinical practice, including patients who, unfortunately, often are excluded from clinical trials. When interpreting the results, it is important to remember that MoCA is a screening tool for cognitive impairment, and the need for further diagnostic inquiries should always be considered. Additionally, it should be noted that we did not use parallel versions of the MoCA test. Thus, a practice effect cannot be ruled out. An objection might be that ECOG PS was not included in the regression model. The number of physical impairments was preferred, as it combines several objective measures of functional status. ECOG PS is observer-dependent and important prognostic information may be lost when applied to older patients [26,59,60]. Furthermore, we did not collect data on psychotropic medications, which might affect cognitive function more than other drugs.

## 5. Conclusions

Compared to age-, gender-, and education-matched cognitively healthy controls, MoCA revealed cognitive impairment in 37.9% of patients ≥65 years referred to RT, implying that CRCI is a clinically relevant problem. Older age, lower education, and physical impairments were independently associated with reduced cognition prior to RT. Four groups with distinct cognitive trajectories ranging from good to very poor were identified, and their baseline characteristics suggested a corresponding range from fit to frail. Except for the very poor group, where a cognitive decline was registered, the remaining trajectories were mainly stable, indicating good tolerance for RT, irrespective of pre-treatment cognitive function. Assessing cognitive function before RT is a prerequisite, and special attention should be given to the oldest and those with other geriatric problems, especially physical impairments.

## Figures and Tables

**Table 1:** Baseline patient characteristics and factors with potential influence on baseline MoCA scores, in total and according to groups with distinct MoCA score trajectories.

	Total *N* = 298	Very Poor Group *N* = 19	Poor Group *N* = 24	Fair Group *N* = 113	Good Group *N* = 142	*p*-Value
Age Mean (SD) Gender, *n* (%) Male Female Education, *n* (%) (1 missing) Compulsory Secondary College or university Comorbidity, CCI Mean (SD) Number of daily medications Mean (SD) Geriatric depression scale ≥5, *n* (%) No Yes	73.6 (6.3) 157 (52.7) 141 (47.3) 90 (30.3) 120 (40.4) 87 (29.3) 1.1 (1.3) 5.4 (3.6) 236 (79.2) 62 (20.8)	77.7 (7.6) 12 (63.2) 7 (36.8) 6 (33.3) 11 (61.1) 1 (5.6) 1.5 (1.4) 8.7 (4.4) 12 (63.2) 7 (36.8)	76.3 (6.4) 9 (37.5) 15 (62.5) 17 (70.8) 5 (20.8) 2 (8.3) 1.4 (1.5) 7.2 (4.0) 17 (70.8) 7 (29.2)	74.7 (6.4) 73 (64.6) 40 (35.4) 42 (38.2) 44 (40.0) 24 (21.8) 1.3 (1.6) 5.7 (3.7) 88 (77.9) 25 (22.1)	71.8 (5.5) 63 (44.4) 79 (55.6) 25 (17.6) 58 (40.8) 59 (41.5) 0.8 (1.0) 4.5 (2.9) 119 (83.8) 23 (16.2)	**<0.001** ^2^ **0.004** ^1^ **<0.001** ^1^ **0.003** ^2^ **<0.001** ^2^ 0.115 ^1^
Number of physical impairments Mean (SD) (3 missing) Fatigue Mean (SD) (3 missing) RT treatment intent, *n* (%) Curative Palliative Previous cancer treatment, *n* (%) Endocrine therapy Other systematic cancer therapy Cancer surgery/RT Brain cancer/brain metastases, *n* (%) No Yes	1.3 (1.4) 37.4 (25.3) 162 (54.4) 136 (45.6) 57 (19.1) 90 (30.2) 182 (61.1) 282 (94.6) 16 (5.4)	3.2 (1.6) ^3^ 45.1 (24.3) 3 (15.8) 16 (84.2) 1 (5.3) 8 (42.1) 10 (52.6) 16 (84.2) 3 (15.8)	1.9 (1.8) 38.9 (27.6) 13 (54.2) 11 (45.8) 7 (29.2) 5 (20.8) 16 (66.7) 22 (91.7) 2 (8.3)	1.5 (1.5) ^4^ 38.2 (26.7) 47 (41.6) 66 (58.4) 27 (23.9) 33 (29.2) 60 (53.1) 108 (95.6) 5 (4.4)	0.8 (0.9) 35.5 (23.9) 99 (69.7) 43 (30.3) 22 (15.5) 44 (31.0) 96 (67.6) 136 (95.8) 6 (4.2)	**<0.001** ^2^ 0.449 ^2^ **<0.001** ^1^ 0.079 ^1^ 0.499 ^1^ 0.091 ^1^ 0.169 ^1^

Abbreviations: CCI, Charlson Comorbidity Index; ECOG PS, Eastern Cooperative Group performance status; Gy, Grey; *p*-value represents comparison of four groups, and *p*-values marked with bold indicate statistically significant differences. ^1^ χ^2^-test, ^2^ ANOVA, ^3^ One missing, ^4^ Two missing.

**Table 2:** MoCA domain scores at baseline (*n* = 298).

MoCA Domains	Maximum Score Possible	Mean Score	Standard Deviation	% with Less than Maximum Score
Visuospatial abilities	5	3.8	1.3	65.1
Naming of objects	3	2.9	0.4	9.4
Attention and concentration	6	5.2	1.1	46.6
Language	3	2.1	0.8	68.1
Abstraction	2	1.3	0.7	59.1
Working memory	5	2.2	1.6	91.9
Orientation to time and place	6	5.8	0.7	13.8

**Table 3:** Characteristics of MoCA test completers and non-completers at 16 weeks after RT.

Total (*n* = 278 ^a^)	Completers (*n* = 167)	Non-Completers (*n* = 111)	*p*-Value ^b^	Non-Completers, Deceased (*n* = 36)	Non-Completers, Alive (*n* = 75)
Baseline MoCA score, mean (SD) Age, mean (SD) Gender, *n* (%) Male Female Education, *n* (%) (1 missing) Compulsory Secondary College or university Comorbidity, CCI, mean (SD) Number of daily medications, mean (SD) Geriatric depression scale ≥5, *n* (%) No Yes	24.7 (3.3) 72.9 (5.9) 81 (48.5) 86 (51.5) 48 (28.7) 68 (40.7) 51 (30.5) 1.0 (1.3) 5.0 (3.5) 136 (81.4) 31 (18.6)	22.9 (4.1) 74.2 (6.7) 63 (56.8) 48 (43.2) 33 (29.7) 47 (42.3) 30 (27.0) 1.2 (1.5) 6.1 (3.7) 82 (73.9) 29 (26.1)	**˂0.001 ^c^** 0.107 ^c^ 0.177 ^d^ 0.843 ^d^ 0.246 ^c^ **0.020**^c^ 0.133 ^d^	21.9 (4.5) 74.2 (7.1) 26 (72.2) 10 (27.8) 12 (33.3) 15 (41.7) 9 (25.0) 1.6 (1.4) 7.6 (3.4) 26 (72.2) 10 (27.8)	23.4 (3.8) 74.2 (6.5) 37 (49.3) 38 (50.7) 21 (28.0) 32 (42.7) 21 (28.0) 1.0 (1.5) 5.3 (3.7) 56 (74.7) 19 (25.3)
Number of physical impairments, mean (SD), (3 missing) Fatigue, mean (SD) (3 missing) RT treatment intent, *n* (%) Curative Palliative Previous cancer treatment, *n* (%) Endocrine therapy Other systematic cancer therapy Cancer surgery/RT Cancer/metastases in the brain, *n* (%) No Yes	0.9 (1.1) 34.3 (23.9) 111 (66.5) 56 (33.5) 38 (22.8) 42 (25.1) 115 (68.9) 162 (97.0) 5 (3.0)	1.9 (1.7) 43.1 (27.2) 40 (36.0) 71 (64.0) 15 (13.5) 42 (37.8) 56 (50.5) 101 (91.0) 10 (9.0)	**˂0.001 ^c^** **0.005 ^c^** ˂0.001 ^d^ 0.163 ^d^ **0.024 ^d^** **0.002 ^d^** **0.030 ^d^**	2.5 (1.6) 58.7 (22.8) 2 (5.6) 34 (94.4) 4 (11.1) 19 (52.8) 16 (44.4) 28 (77.8) 8 (22.2)	1.6 (1.6) 35.7 (26.0) 38 (50.7) 37 (49.3) 11 (14.7) 23 (30.7) 40 (53.3) 73 (97.3) 2 (2.7)

^a^ Accounting for protocol exceptions (*n* = 20), i.e., patients recruited from municipalities that did not participate in performing the mGA during follow-up. Of the 39 patients that were deceased by 16 weeks after RT, 3 were recruited from such municipalities. ^b^ *p*-value represents comparison of MoCA completers and all non-completers, irrespective of cause, 16 weeks after RT. ^c^ Independent samples *t*-test. ^d^ χ^2^-test. *p*-values marked with bold indicate statistically significant differences.

**Table 4:** Results of linear regression analyses investigating factors associated with baseline MoCA scores, (*n* = 294).

Covariate	Unadjusted Models		Adjusted Model	
	RC (95% CI)	*p*-Value	RC (95% CI)	*p*-Value
**Age** **Gender,** Female **Education,** n Compulsory Secondary College or university **Comorbidity,** CCI **Number of daily medications** **Geriatric depression scale ≥5**	−0.22 (−0.28; −0.16) 0.72 (−0.12; 1.57) 0 1.42 (0.47; 2.37) 3.35 (2.32; 4.38) −0.63 (−0.94; −0.33) −0.37 (−0.48; −0.25) −1.48 (−2.51; −0.45)	**<0.001** 0.094 **0.004** **<0.001** **<0.001** **<0.001** **0.005**	−0.13 (−0.19; −0.07) 0.28 (−0.49; 1.05) 0 0.73 (−0.11; 1.57) 2.41 (1.50; 3.33) 0.02 (−0.30; 0.33) −0.11 (−0.24; 0.02) −0.26 (−1.25; 0.74)	**<0.001** 0.479 0.089 **<0.001** 0.924 0.107 0.613
**Number of physical impairments** **Fatigue** **RT treatment intent,** Palliative **Previous cancer treatment** Endocrine therapy Other systematic cancer therapy Cancer surgery/RT **Cancer/metastases in the brain**	−1.23 (−1.49; −0.97) −0.02 (−0.04; −0.003) −1.84 (−2.67; −1.02) −0.12 (−1.20; 0.96) 0.43 (−0.49; 1.36) 0.89 (0.03; 1.76) −0.98 (−2.85; 0.88)	**<0.001** **0.021** **<0.001** 0.822 0.360 **0.043** 0.300	−0.82 (−1.16; −0.48) 0.01 (−0.004; 0.03) −0.54 (−1.41; 0.33) 0.14 (−0.81; 1.08) 0.55 (−0.32; 1.42) 0.09 (−0.69; 0.87) −0.06 (−1.70; 1.58)	**<0.001** 0.141 0.223 0.778 0.216 0.817 0.940

Abbreviations: RC, regression coefficient; CI, confidence interval. *p*-values marked with bold indicate statistically significant differences.

**Table 5:** Results of growth mixture model for MoCA scores, *n* = 298.

	Very Poor *N* = 19 (6.4%)		Poor *N* = 24 (8.1%)		Fair *N* = 113 (37.9%)		Good *N* = 142 (47.7%)	
	RC (SE)	*p*-Value	RC (SE)	*p*-Value	RC (SE)	*p*-Value	RC (SE)	*p*-Value
Intercept Linear Quadratic MoCA ^a^ T0 T1 T2 T3	16.36 (0.60) −0.93 (0.24) 0.05 (0.01) 16.4 14.1 12.5 17.7	<0.001 <0.001 <0.001	20.49 (0.58) 0.02 (0.05) 20.5 20.6 20.7 20.9	<0.001 0.641	22.79 (0.37) 0.41 (0.08) −0.01 (0.004) 22.8 23.9 25.7 26.0	<0.001 <0.001 0.004	26.68 (0.23) 0.43 (0.07) −0.01 (0.003) 26.6 27.6 28.9 29.0	<0.001 <0.001 <0.001
Av.prob.	0.84		0.86		0.79		0.91	

Abbreviations: RC, regression coefficient; SE, standard error; T0, baseline; T1, at RT completion; T2, 8 weeks after RT; T3, 16 weeks after RT. Av.prob, average group probability. ^a^ Predicted mean MoCA values.

## Data Availability

According to Norwegian regulations, research data are confidential due to patient privacy protection. On individual, specific request, anonymized data could be made available.

## Supplementary Materials

The following supporting information can be downloaded at: https://www.mdpi.com/article/10.3390/curroncol29070409/s1. Table S1: Spearman’s rho correlation for factors included in the linear regression models; Figure S1: Second sensitivity analysis, growth mixture model including only patients who completed MoCA at all time points assessed; Table S2: Results of growth mixture model, second sensitivity analysis only including patients who completed MoCA at all time points assessed.
