# Supplementary material for: Cognitive Trajectories in Older Patients with Cancer Undergoing Radiotherapy—A Prospective Observational Study
Source: Curr Oncol. 2022 Jul 21;29(7):5164–78. doi: 10.3390/curroncol29070409 (PMC9317354; doi:10.3390/curroncol29070409)
Supplement: Supplementary file 1 [file curroncol-29-00409-s001.zip › curroncol-1802381-supplementary.pdf]

Supplementary Table S1. Spearman´s rho correlation for factors included in the linear regression models.

Spearman's rho		2	3	4	5	6	7	8	9	10	11	12	13	14
MoCA	1	-0.05	0.07	0.14	-0.36	-0.25	-0.05	-0.12	-0.34	0.14	0.37	-0.24	-0.29	-0.14
Endocrine therapy	2		-0.01	-0.27	-0.01	0.06	-0.04	0.04	0.14	-0.20	0.02	-0.03	0.10	0.01
Other systemic cancer therapy	3			0.11	0.10	0.32	-0.03	0.36	-0.05	0.08	-0.07	-0.02	0.11	0.16
Cancer surgery/RT	4				-0.10	-0.13	0.07	0.01	-0.14	0.31	0.01	-0.14	-0.11	0.06
Number of physical impairments	5					0.44	0.19	0.45	0.21	-0.01	-0.13	0.27	0.51	0.39
RT treatment intent	6						0.26	0.31	0.21	-0.21	-0.06	0.19	0.29	0.16
Cancer/metastases in the brain	7							0.05	-0.06	-0.05	0.04	-0.05	0.01	0.14
Fatigue	8								0.11	0.13	-0.04	0.20	0.40	0.45
Age	9									-0.12	-0.14	0.18	0.25	0.05
Gender	10										-0.01	-0.20	-0.05	0.05
Educational level (three categories)	11											-0.18	-0.18	-0.05
Comorbidity, CCI	12												0.56	0.08
Number of daily medications	13													0.27
Geriatric depression scale ≥5	14													

Supplementary Figure S1. Second sensitivity analysis, growth mixture model including only patients who completed MoCA at all time points assessed, n=113.

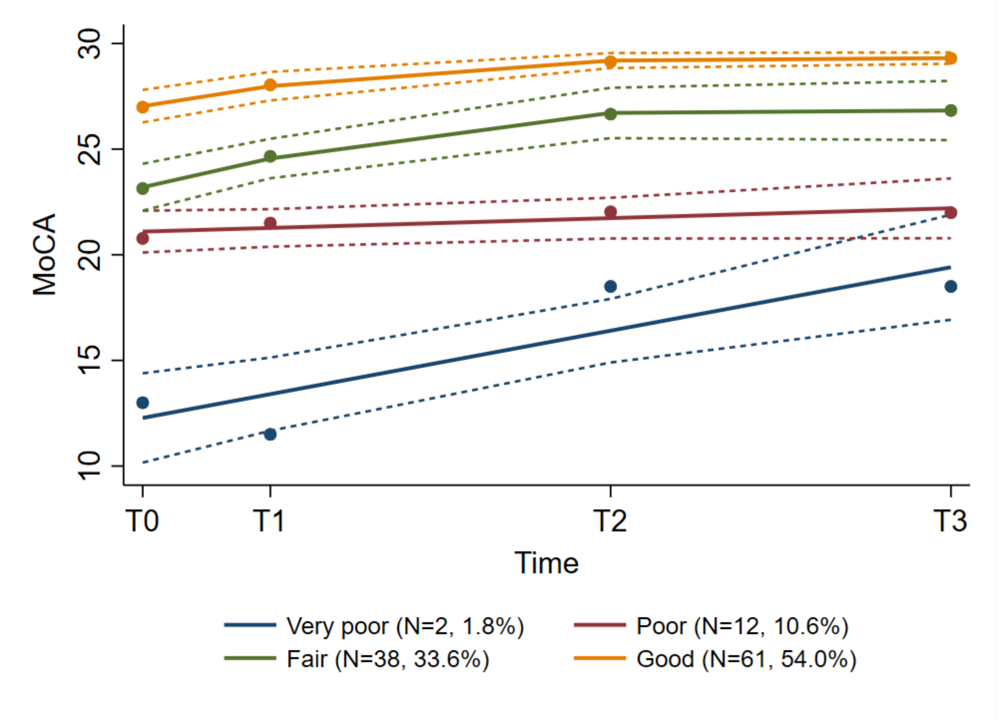

Supplementary Table S2. Results of growth mixture model, second sensitivity analysis only including patients who completed MoCA at all time points assessed.

	Very poor N=2[a] (1.8%)		Poor N=12 (10.6%)		Fair N=38 (33.6%)		Good N=61 (54.0%)	
	RC (SE)	p-value	RC (SE)	p-value	RC (SE)	p-value	RC (SE)	p-value
Intercept	12.28 (1.08)	<0.001	21.10 (0.50)	<0.001	23.20 (0.56)	<0.001	27.10 (0.29)	<0.001
Linear	0.38 (0.10)	<0.001	0.06 (0.04)	0.182	0.50 (0.10)	<0.001	0.40 (0.08)	<0.001
Quadratic					-0.02 (0.005)	0.002	-0.01 (0.004)	0.002
Av.prob.	0.99		0.92		0.86		96	

Abbreviations: RC, regression coefficient; SE, standard error; T0, baseline; T1, at RT completion; T2, 8 weeks after RT; T3, 16 weeks after RT. Av.prob, average group-probability. [a]The very poor group contains only two patients, but none of the applied statistical criteria (except for reasonable group size) suggests that this group could be merged with another one.
